# Parkinson's Disease Diagnosis Using Neostriatum Radiomic Features Based on T2-Weighted Magnetic Resonance Imaging

**DOI:** 10.3389/fneur.2020.00248

**Published:** 2020-04-08

**Authors:** Panshi Liu, Han Wang, Shilei Zheng, Fan Zhang, Xianglin Zhang

**Affiliations:** ^1^Department of Radiology, First Affiliated Hospital of Jinzhou Medical University, Jinzhou, China; ^2^Medical Imaging Center, Taian Central Hospital, Taian, China; ^3^Department of Neurology, First Affiliated Hospital of Jinzhou Medical University, Jinzhou, China

**Keywords:** Parkinson's disease, diagnosis, radiomics, neostriatum, T2-weighted imaging

## Abstract

**Background:** Parkinson's disease (PD) is a neurodegenerative disease in which the neostriatum, including the caudate nucleus (CN) and putamen (PU), has an important role in the pathophysiology. However, conventional magnetic resonance imaging (MRI) lacks sufficient specificity to diagnose PD. Therefore, the study's aim was to investigate the feasibility of using a radiomics approach to distinguish PD patients from healthy controls on T2-weighted images of the neostriatum and provide a basis for the clinical diagnosis of PD.

**Methods:** T2-weighted images from 69 PD patients and 69 age- and sex-matched healthy controls were obtained on the same 3.0T MRI scanner. Regions of interest (ROIs) were manually placed at the CN and PU on the slices showing the largest respective sizes of the CN and PU. We extracted 274 texture features from each ROI and then used the least absolute shrinkage and selection operator regression to perform feature selection and radiomics signature building to identify the CN and PU radiomics signatures consisting of optimal features. We used a receiver operating characteristic curve analysis to assess the diagnostic performance of two radiomics signatures in a training group and estimate the generalization performance in the test group.

**Results:** There were no significant differences in the demographic and clinical characteristics between the PD patients and healthy controls. The CN and PU radiomics signatures were built using 12 and 7 optimal features, respectively. The performance of the two radiomics signatures to distinguish PD patients from healthy controls was good. In the training and test groups, the AUCs of the CN radiomics signatures were 0.9410 (95% confidence interval [CI]: 0.8986–0.9833) and 0.7732 (95% CI: 0.6292–0.9173), respectively, and the AUCs of the PU radiomics signature were 0.8767 (95% CI: 0.8066–0.9469) and 0.7143 (95% CI: 0.5540–0.8746), respectively. Vertl_GlevNonU_R appeared simultaneously in both the CN and PU radiomics signatures as an optimal feature. A *t*-test analysis revealed significantly higher levels of texture values of the CN and PU in the PD patients than healthy controls (*P* < 0.05).

**Conclusion:** Neostriatum radiomics signatures achieved good diagnostic performance for PD and potentially could serve as a basis for the clinical diagnosis of PD.

## Introduction

Parkinson's disease (PD) is a progressive neurodegenerative disease characterized by rigidity, tremor, slowed movements, and other non-motor symptoms. PD affects 2–3% of elderly people >65 years old worldwide and has a significant impact on patients' quality of life (1). PD mainly causes degeneration of the dopaminergic neurons of the nigrostriatal system (2). As part of the nigrostriatal system, the neostriatum includes both the caudate nucleus (CN) and putamen (PU) (3). Abnormal deposition of cytoplasmic inclusions (Lewy bodies) containing a-synuclein and ubiquitin in the neostriatum has been proven pathologically (4). Additionally, iron deposition in the brain, including the neostriatum, has been proposed as having an important role in the pathophysiology of PD (5, 6). Additionally, converging evidence has demonstrated the existence of striatal microstructural changes (7, 8).

The clinical diagnosis of PD essentially relies on a set of clinical presentations that do not provide high accuracy (9). In neuroimaging, based on the above-mentioned pathophysiological changes in PD, some advanced magnetic resonance imaging (MRI) sequences, such as neuromelanin-sensitive MRI (10) and quantitative susceptibility mapping (11), have been reported to be useful for diagnosis of PD. Resting-state functional MRI and diffusion tensor imaging (DTI) also have demonstrated abnormalities in the neostriatum in PD patients (7, 8, 12). Unfortunately, the limitations of these techniques, such as complicated sequences and time-consuming procedures, have hindered their clinical application. As a basic radiological examination, conventional MRI is widely used in clinical neurology. However, conventional MRI only serves to exclude underlying pathologies (e.g., cerebrovascular disease) and lacks specificity in diagnosis (13).

Radiomics, which use mathematical methods to examine a large set of texture features and extract mineable high-dimensional data from the texture features, can provide non-visual information of medical images, such as microstructural alterations and even pathological changes (14). The “texture features” are the interrelationships of image pixel gray-levels and patterns, which are hard to see directly by radiologists. According to the calculation method, these could be divided into first-, second-, or higher-order features. After texture feature extraction, radiomics use machine learning or advanced statistical methods to analyze the high-dimensional feature data to identify the optimal features. Finally, a radiomics signature, a clinical classifier model consisting of the optimal features, was built (14). In the early years of radiomics, this new approach has been widely applied in oncology fields and has shown potential benefits for tumor grading and pathological classification (15, 16). However, given its power in capturing the microstructural changes in tissues and its correlation with clinical endpoints (17) and age progression (18, 19), the use of radiomics is expected to increase in neurodegenerative disorders (20). Presently, radiomics has been applied to the diagnosis of neurodegenerative diseases, including Alzheimer's disease (AD), amyotrophic lateral sclerosis, and Machado–Joseph disease with conventional MRI (21–25), which have similar pathological changes with PD, such as atrophy, abnormal proteins, or iron deposition in many brain regions. In a longitudinal study, radiomics successfully detected microstructural changes in invisible normal-appearing white matter on conventional T2 fluid-attenuated inversion-recovery (FLAIR) images (18). In addition, recent studies indicated that radiomics features derived from DAT SPECT images can serve as a biomarker for PD progression tracking (26, 27). Notably, a previous texture analysis study demonstrated that texture features based on T2-weighted imaging (T2WI) differed between PD patients and healthy controls in many brain regions (28). However, there is no published study that investigated the diagnosis of PD on conventional MRI by using a radiomics approach.

Therefore, the study aim was to investigate the feasibility of using a radiomics approach for discrimination of PD patients from healthy controls on T2WI, which may provide a basis for the clinical diagnosis of PD and guide management for precision medicine. To this end, we developed two T2WI-based radiomics signatures, each consisting of optimal features for either CN or PU, and then estimated the generalization performance in the test group by using receiver operating characteristic (ROC) analysis.

## Materials and Methods

### Participants

This retrospective study was approved by the Ethics Committee at the First Affiliated Hospital of Jinzhou Medical University, and the requirement for written informed consent was waived by the Ethics Committee. MRI and clinical data were retrospectively and anonymously collected from the Picture Archiving and Communication System and medical records of our hospital. All the data were anonymized and de-identified prior to analysis. The inclusion criteria for PD patients was a clinical diagnosis of idiopathic PD per the UK Parkinson Society Brain Bank criteria (29). The exclusion criteria for PD patients were history of other neurological and psychiatric diseases, secondary or atypical Parkinsonism, history of alcohol and/or drug abuse, and history of head injury. The healthy controls with age and sex matched to the PD patients were recruited from the medical examination center of the hospital. All healthy controls had no history of any neurological or psychiatric disorders, alcohol and/or drug abuse, or head injury. All participants underwent conventional MRI examinations with T2WI on the same 3.0T MRI scanner. The T2W images from each participant were evaluated for artifacts that may have affected the feature extraction. Satisfactory image quality for further analysis was obtained for all participants. Sixty-nine PD patients (40 male; 72.4 ± 9.1 years) and 69 healthy controls (35 male; 70.09 ± 5.6 years) were reviewed, according to the above criteria, from February 2017 to December 2018. The PD patients and healthy controls were randomly allocated to training (*n* = 48) and test groups (*n* = 21) at a proportion of 7:3.

### MRI Acquisition

**Figure 1:**
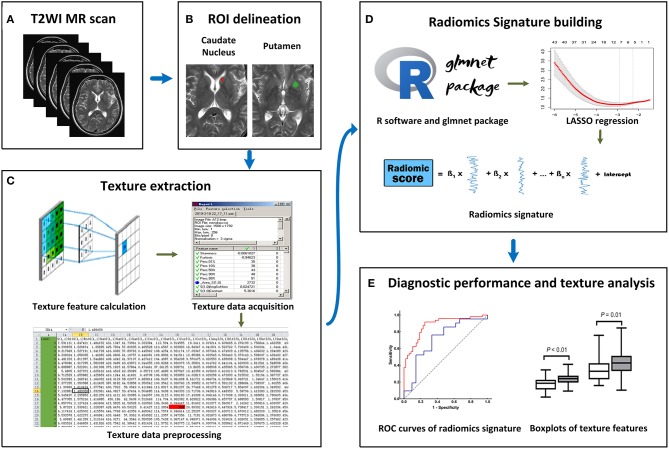
Workflow of this study. **(A)** All T2-weighted imaging (T2WI) images were acquired on the same MRI scanner. **(B)** Regions of interest (ROIs) were manually placed on the bilateral caudate nucleus (CN) and putamen (PU). **(C)** A total of 274 radiomics features were extracted from each ROI. **(D)** On the R software platform with the glmnet package installed, least absolute shrinkage and selection operator (LASSO) regression was used to optimal features selection and radiomics signature building. **(E)** Receiver operating characteristic (ROC) curves were used to evaluate classification performance, and *t*-test analysis and box plots were used to compare the co-occurring optimal feature values.

All MRI images were acquired on the same 3.0T Verio Siemens scanner (Siemens, Erlangen, Germany) equipped with an eight-channel head coil. The acquisition parameters were as follows: (1) axial T1 weighted scan (T1WI) [repetition time (TR)/echo time (TE)/inversion time (TI), 2,000/9/860 ms]; (2) axial T2WI (TR/TE, 6,000/96 ms); (3) axial FLAIR (TR/TE/TI, 8,500/94/2,440 ms); These three sequences shared the following parameters: field of view (FOV), 240 × 204 mm; matrix, 320 × 240 pixels; section thickness, 5 mm; (4) sagittal T1WI (TR/TE/TI, 2,000/9/860 ms; FOV, 240 × 216 mm; matrix, 320 × 240 pixels; section thickness, 5.5 mm) (Figure 1A).

### Image Selection and Region of Interest (ROI) Delineation

The T2W images were used for feature extraction, and T1W and FLAIR images were used as anatomical references for the ROI delineation and placement. The T2W image quality was evaluated, and the slices for feature extraction were determined by two neurological radiologists (SZ and XZ) who had 9 and 28 years of experience, respectively. To extract the texture features more comprehensively and reduce the partial volume effect due to slice thickness, two slices showing the largest respective sizes of CN and PU in each participant were selected for feature extraction. In cases of disagreement of image selection, consensus was reached through discussion (the outcome of the consensus between the two radiologists can be found in Supplementary Table 1).

ROI delineation and texture extraction were performed by using the available MaZda software (MaZda Version 4.6; Technical University of Lodz, Poland). To minimize confounding effects in the image, image gray-level intensity normalization was performed by discarding the image intensities not within μ ± 3δ (μ: mean of gray-level value, δ: standard deviation of gray-level value) (30).

Since different ROI sizes can affect the results of texture extraction (31), to ensure the stability of texture extraction, an ROI was delineated as a rotundity with the same size. In addition, the ROI cannot exceed or coincide the edges of the CN or PU to ensure that it is within the region of the CN or PU. Further, two ROIs were exported as ^*^.roi files with a size of 1,257 pixels and 2,552 pixels for the CN and PU, respectively. Next, we imported the selected CN and PU slices in the MaZda software and then loaded the respective ^*^.roi files. The ROIs were manually placed on the bilateral CN and PU by two neurological radiologists (SZ and XZ, respectively), (Figure 1B). Radiologist SZ placed the ROIs again within a 2 weeks interval.

### Texture Extraction

The MaZda software “Analysis” module was used for texture extraction. A total of 274 texture features were extracted from each ROI, and the results were exported as ^*^.sel files (Figure 1C).

### Radiomics Signatures Building

LASSO regression with filter, which is a sparse learning method suitable for high-dimensional data (32), was used to optimal feature selection and radiomics signatures building. LASSO regression reduced the penalty term lambda to set the coefficients of diagnostic-unrelated features to zero and retain optimal features with non-zero coefficients. To select optimal features in LASSO regression, we employed 10-fold cross-validation with binomial deviance following minimization criteria to determine the optimal penalty term lambda (33). With lambda determined, the radiomics signature was generated by the multivariate LASSO-logistic regression analysis. To be specific, the radiomics signature was a linear equation consisting of an intercept and optimal features multiplied by their respective coefficients. For each participant in the training group, we substituted the optimal feature values into the equation to obtain their radiomics score (Figure 1D).

### Validation of the Radiomics Signatures

The ROC curve is widely used to evaluate performance of supervised classification (34) and is a common method for the measurement of radiomics signature performance in the field of radiomics research (35). The classification performance of our radiomics signature was measured by a ROC curve and the corresponding AUC in this study, and sensitivity and specificity were also calculated. We plotted the ROC curve of the radiomics score and calculated the AUC, sensitivity, and specificity in the training group. To estimate the generalization performance of our radiomics signatures, we used the same image selection and ROI delineation methods to extract the texture features of the CN and PU in the test group. In the same way, the radiomics score was calculated for each participant in the test group using our radiomics signatures by substituting optimal feature values into the equation. The ROC curve of the test group's radiomics score was plotted to show the generalization performance of the signature, and then the AUC, sensitivity, and specificity were calculated (Figure 1E).

### Statistical Analysis

All statistical analyses were performed by using SPSS statistical software (Version 20.0; SPSS Inc., Chicago, IL, USA), R software (Version 3.4.3; R Foundation for Statistical Computing, Vienna, Austria), and GraphPad Prism (Version 8.0.2; GraphPad Software, Inc., San Diego, CA). The differences in age, sex, duration of disease, and modified Hoehn–Yahr stage between the study groups were investigated by performing the Chi-square, Student's *t*, and Mann–Whitney *U* tests. LASSO regression was performed by using the “glmnet” package in R software. The Hosmer–Lemeshow test was performed to evaluate the goodness-of-fit of radiomics signatures. ROC curves were used to evaluate the diagnostic classification performance of radiomics signatures. The differences in co-occurring optimal feature values between the PD patients and healthy controls were analyzed by using Student's *t*-test. The inter- and intraobserver agreements of feature extraction reproducibility were evaluated by inter- and intra-class correlation coefficients (ICCs). The interobserver ICC was calculated by using each texture feature for agreement between SZ and XZ, and the intraobserver ICC was calculated by using each texture feature for agreement between two performances by SZ. The threshold for statistical significance was set to *P* < 0.05, and all reported *P*-values were two-sided.

## Results

### Demographics and Clinical Characteristics

The demographic and clinical characteristics of the participants for the training and test group are shown in Table 1. We found no significant differences in age and sex between the healthy controls and PD patients in the training and test groups. Among the PD patients, there were no significant differences in demographic characteristics, modified Hoehn–Yahr stage, and duration of disease between the two groups.

**Table 1:** Comparison of demographic and clinical characteristics between patients with PD and healthy controls in the training and test groups.

**Characteristics**	**Training group**			**Test group**			**HC-Training vs. HC-Test** ***P* value**	**PD-Training vs. PD-Test** ***P*-value**
	**HC** **(*n* = 48)**	**PD** **(*n* = 48)**	***P-*value**	**HC** **(*n* = 21)**	**PD** **(*n* = 21)**	***P-*value**		
Age (years, mean ± SD)	70.48 ± 5.43	72.91 ± 9.89	0.124^a^	69.19 ± 5.94	71.38 ± 9.54	0.377^a^	0.381^a^	0.544^a^
Gender (male/female)	25/23	27/21	0.838^b^	10/11	13/8	0.535^b^	0.937^b^	0.863^b^
Duration of disease^#^ (years, mean ± SD)		4.75 ± 2.92			3.71 ± 2.19			0.111^a^
Modified H&Y stage(median, IQRor mean ± SD)		2.5 (2.0, 3.0)			2.52 ± 0.97			0.711^c^

*HC, healthy controls; HC-Training, healthy controls in the training group; HC-Test, healthy controls in the test group; PD, patients with Parkinson's disease; PD-Training, Parkinson's disease patients in the training group; PD-Test, Parkinson's disease patients in the test group; SD, standard deviation; Modified H&Y stage, modified Hoehn–Yahr stage; IQR, interquartile range*.

# *The mean disease duration of PD patients, defined as the time between when a patient subjectively noticed his or her first symptoms and the moment of assessment*.

a Two-sample Student's t-test;

b Chi-square test;

c *Mann–Whitney U-test*.

### Feature Extraction and Radiomics Signature Building

**Figure 2:**
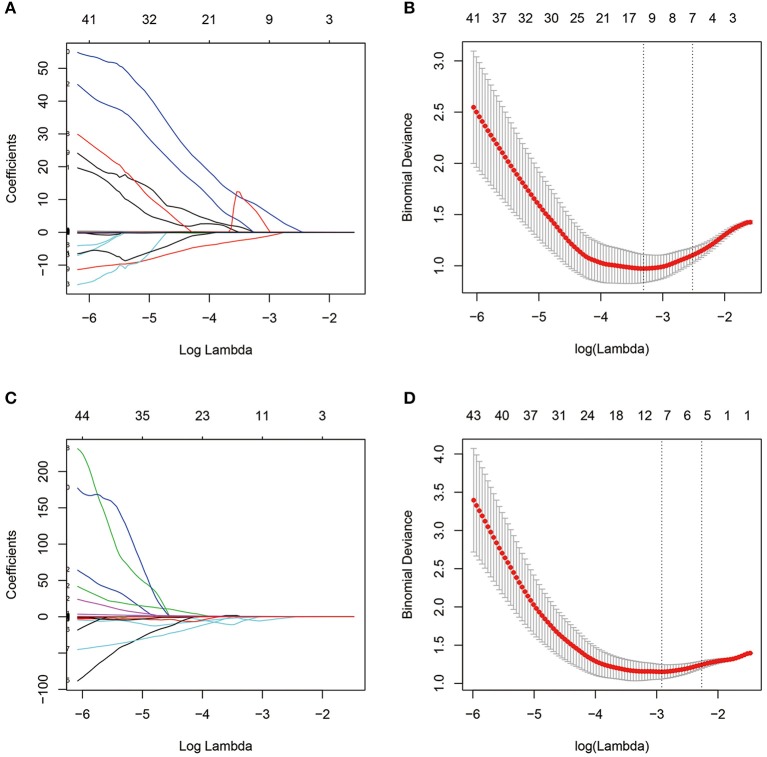
Dimensionality reduction with LASSO regression. **(A,C)** The coefficients of texture features, represented by each colored line, were plotted vs. log (lambda) in the caudate nucleus (CN) and putamen (PU) radiomics signatures, respectively. **(B,D)** The binomial deviances were plotted vs. log (lambda) in the CN and PU radiomics signatures, respectively. Using 10-fold cross-validation, the red points indicate average values of deviance for each lambda, and the left and right dotted vertical lines correspond to lambda in the minimum criteria and the one standard error of the minimum criteria, respectively.

A total of 274 texture features were extracted from each ROI. The interobserver ICCs ranged from 0.745 to 0.901, and the intraobserver ICCs ranged from 0.776 to 0.924, suggesting favorable reproducibility of feature extraction. Figure 2 shows the dimensionality reduction after LASSO regression. Figures 2A,C shows the trace plots of the texture feature coefficients fit by LASSO. As shown, more coefficients of diagnostic-unrelated features were set to zero with increasing value of the penalty term lambda, which left fewer optimal features with non-zero coefficients in the equation. Figures 2B,D shows the determination of the penalty term lambda based on 10-fold cross-validation. To determine the minimization criteria of binomial deviance, we picked the optimized lambda at the left dotted vertical lines: a lambda value of 0.037 with log (lambda) = −3.310 and a lambda value of 0.052 with log (lambda) = −2.963 were selected for the CN and PU radiomics signatures, respectively. After the dimensionality reduction, there were 12 and seven optimal features with non-zero coefficients remaining in the CN and PU radiomics signatures, respectively (Table 2). All optimal features were derived from image histogram, run-length matrix, and co-occurrence matrix texture features (Table 2). Finally, two neostriatum radiomics signatures were built on the basis of LASSO-logistic regression analysis; the optimal features, coefficients, and intercept are shown in Table 2.

**Table 2:** Optimal features after the dimensionality reduction.

**Optimal features**		**Coefficient/Intercept**
**CN radiomics signature**		−15.160413946
Histogram	Perc.50%_R	−0.027683304
Run-length matrix	Vertl_GlevNonU_R^*^	0.112951984
	Vertl_GlevNonU_L	0.019889353
	45dgr_RLNonUni_L	0.001556838
	135dr_RLNonUni_R	0.005421572
Co-occurrence matrix	S (1,0)Correlat_L	8.118174099
	S (1,0)Correlat_R	9.221141623
	S (0,1)SumVarnc_R	0.001540649
	S (1,1)Contrast_R	−2.039856941
	S (1,−1)Contrast_L	−0.142200691
	S (4,−4)InvDfMom_L	0.750813907
	S (5,5)InvDfMom_R	0.462795637
**PU radiomics signature**		2.705271e+00
Histogram	Kurtosis_L	4.774812e−01
	Perc.01%_R	−3.950184e−02
Run-length matrix	Vertl_GlevNonU_R^*^	1.979927e−02
	45dgr_RLNonUni_L	4.756729e−06
Co-occurrence matrix	S (2,-2)Contrast_L	−1.047061e−02
	S (5,0)InvDfMom_L	−4.534430e+00
	S (0,5)Contrast_R	−5.781975e−03

*CN, caudate nucleus; PU, putamen*.

* *Appeared simultaneously in two neostriatum radiomics signatures*.

### Texture Feature Analysis

**Figure 3:**
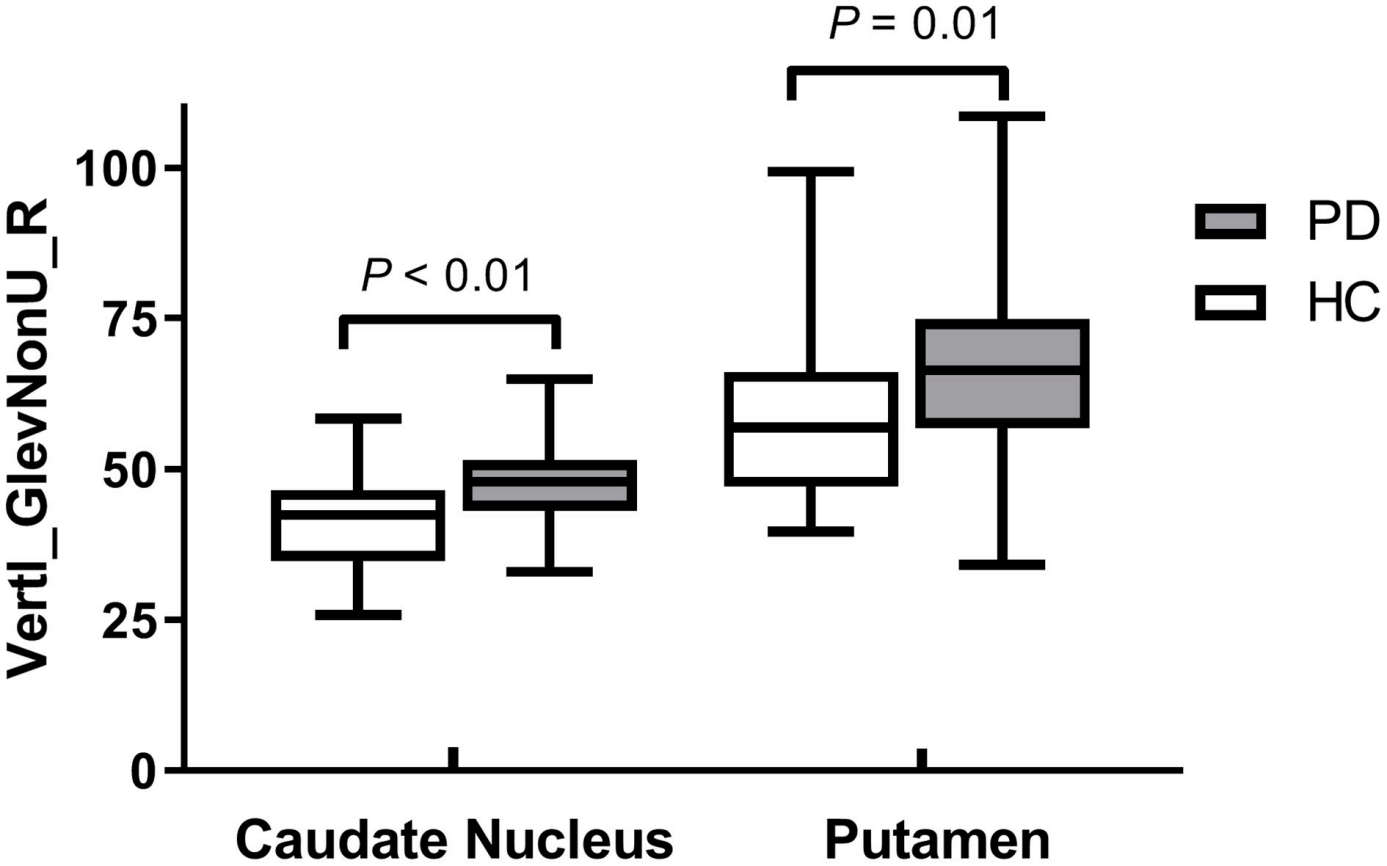
Box plots of optimal feature that appeared simultaneously in two radiomics signatures.

Vertl_GlevNonU_R, a second-order run-length matrix texture feature, appeared simultaneously in both the CN and PU signatures as an optimal feature (Table 2, marked with an asterisk), which means this feature has high diagnostic value in both CN and PU. The *t*-test analysis revealed significantly higher levels of texture values for both the CN and PU in the PD patients than in the healthy controls (*P* < 0.05) (Figure 3).

### Diagnostic Performance of Two Neostriatum Radiomics Signatures

**Figure 4:**
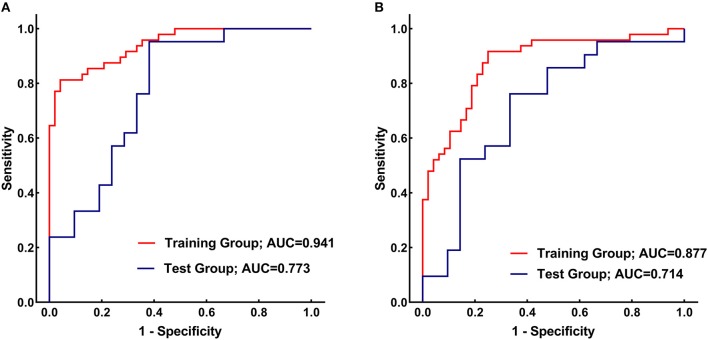
**(A)** Receiver operating characteristic (ROC) curves of the caudate nucleus (CN) radiomics signature for distinguishing between Parkinson's disease (PD) patients and healthy controls in the training (red) and test (blue) groups. **(B)** ROC curves of the putamen (PU) radiomics signature for distinguishing between PD patients and healthy controls in the training (red) and test (blue) groups.

Figure 4A presents the performance of the CN radiomics signature for distinguishing PD from healthy controls in the two groups. In the training group, the AUC, sensitivity, and specificity were 0.9410 (95% confidence interval [CI]: 0.8986–0.9833), 81.25, and 95.83%, respectively. In the test group, the AUC, sensitivity, and specificity were 0.7732 (95% CI: 0.6292–0.9173), 95.24, and 61.90%, respectively. The Hosmer–Lemeshow test showed an acceptable goodness-of-fit of CN radiomics signature in the training and test groups (*P* = 0.404, 0.591, respectively).

Figure 4B shows the performance of the PU radiomics signature for distinguishing PD from healthy controls in the two groups. In the training group, the AUC, sensitivity, and specificity were 0.8767 (95% CI: 0.8066–0.9469), 91.67, and 75.00%, respectively. In the test group, the AUC, sensitivity, and specificity were 0.7143 (95% CI: 0.5540–0.8746), 76.19, and 66.67%, respectively. The Hosmer–Lemeshow test showed an acceptable goodness-of-fit of PU radiomics signature in the training and test groups (*P* = 0.285, 0.508, respectively).

## Discussion

Using T2W images with feature extraction and a high-throughput radiomics approach, we showed for the first time that conventional MRI-based radiomics signatures with good diagnostic performance for PD could be built. Our results indicated that neostriatum radiomic features had good potential as diagnostic markers for PD. In addition, we also evaluated the differences in co-occurring optimal feature values between PD patients and healthy controls.

PD is characterized by nigrostriatal degeneration. Although the pathogenesis of PD has not yet been clearly elucidated, iron deposition in the neostriatum has been demonstrated in earlier studies (5, 6). Being paramagnetic, iron can influence T2 relaxation times and, therefore, T2 signals (36–38), which can cause local signal non-uniformity. Moreover, decreases in the T2 signals in the neostriatum have been found in an earlier study (39). Although diagnosis of PD on conventional MRI by measuring the striatum T2 signal is non-specific (40), the local signal non-uniformity might cause changes in the neostriatum textural patterns of T2W images. In addition, pathological and DTI studies have demonstrated the presence of Lewy body deposits and microstructural deficits in the neostriatum of PD patients (4, 7, 8, 41). All of these microstructural changes might be characterized by alteration of textural patterns of T2W images (28) and captured by radiomics approach.

In the current study, according to the calculation method, optimal features (Table 2) can be classified into three major categories: the histogram, co-occurrence matrix, and run-length matrix features (42). Further, these major category features were subclassified into two, four, and two minor categories in the histogram, co-occurrence matrix, and run-length matrix features, respectively. As a first-order texture, the histogram only describes the gray-level distribution in the ROI without considering the neighboring pixels as follows: (1) Kurtosis reflects the shape of a histogram and is used to measure the asymmetry of the ROI (43); (2) The Perc indicates the highest gray-level containing a given percentage of pixels in the image (42). The co-occurrence matrix is based on an estimation of the second-order joint conditional probability density functions [p(x,y)]. Each p(x,y) is the probability of a pair of gray values, x and y, in a specified displacement of an image (44). (1) Contrast describes the local variation presented in an image; (2) Correlation describes the image complexity; (3) SumVarnc measures the spread in the sum of the gray-levels of the pixel-pair distribution; (4) InvDfMom describes the local degree of homogeneity (45). The gray-level run-length matrix is based on computing the number of same gray-level runs of various lengths in a given direction (in general, the vertical, horizontal, and two-diagonal directions) (44). Both texture features, GlevNonU and RLNonUni, are derived from the run-length feature matrix. Therefore, the GlevNonU and RLNonUni features are a measure of a given direction's homogeneity of the pixel gray-level distribution of the underlying tissue, with higher values representing more inhomogeneity within the gray-levels of the run-length matrix (46). We found that the feature Vertl_GlevNonU_R co-occurred in two signatures, as one of the optimal features, and their feature values in both the CN and PU were significantly higher in the PD patients than in the healthy controls. Thus, combined with the pathological basis, we hypothesized that the significantly higher value of Vertl_GlevNonU_R might be related to the more heterogeneous texture patterns caused by iron deposition in the neostriatum in patients with PD.

Radiomics has been successfully applied to neurodegenerative disease studies. In a recent radiomics study by Feng et al. (23), T1W images from 78 patients with AD and 44 healthy controls were used for radiomics analysis. The corpus callosum was segmented manually, and texture features were obtained after extraction from each subject. After LASSO dimensionality reduction, a diagnostic model containing 11 features was established, which achieved an AUC of 0.72 for diagnosing AD. In another AD study, a support vector machine model demonstrated that hippocampal radiomics features could distinguish AD from healthy controls, with an AUC of 0.93 (22). Similar to these previous studies, we also used a general radiomics approach to extract features and build models of brain regions that had undergone microstructural changes that were not yet visible on conventional MRI. Previous studies above and our results suggested that radiomics could be used as biomarkers for neurodegenerative diseases on conventional MRI. Sikio et al. (28) applied texture analysis to PD for the first time and proved that the texture features of PD patients were changed in multiple brain regions (including the neostriatum), and stated that texture analysis could detect microstructural changes in T2W brain MRI images. However, they only extracted a few co-occurrence matrix features without further mining and modeling. In the current study, we extracted multiple categories of texture features and employed LASSO method to build radiomics signatures for diagnosis. As a popular machine-learning algorithm, LASSO is widely used as a high-dimensional data analysis tool in radiomics research because it is designed to avoid overfitting, so it can analyze large sets of texture features with a relatively small sample size (32). To our knowledge, no reported radiomics study has described the establishment of a PD diagnostic model for conventional MRI. This is the first radiomics study to diagnose PD by using conventional MRI.

Our study had several limitations. First, we built CN and PU radiomics signatures, respectively, rather than as a combined radiomics signature because of an insufficient sample size (the number of observations did not match the number of variables in the matrix, which meant that LASSO regression could not be performed in the “glmnet” package). Second, the sample size was insufficient to perfectly train the radiomics signature, leading to some degree of overfitting, which can be reflected in the AUC loss between the training group, and the test group. Therefore, further studies with greater sample size of training data set are necessary to reduce the degree of overfitting and improve the generalization performance. Third, the manual image selection and ROI determination was labor-intensive and time-consuming in the current study, which hinders the large sample size research progress and clinical application of radiomics. As the training sample size increases in future research, it is crucial to develop automatic or semi-automatic image selection and ROI determination algorithms to improve the research feasibility and to reduce inter-operator variability. Forth, along with age growth, a series of physiological changes in the brain also affect the texture features (47). Although there was no statistically significant difference in age ratios between the two groups, we did not achieve a complete 1:1 match, so the effects of this incomplete match on the research results cannot be completely excluded. Lastly, this was a retrospective study in which all subjects were recruited from a single hospital. In the future, a large-sample multi-center study is needed to evaluate the generalization performance and potential for clinical translation of our radiomics signatures. Moreover, the usefulness of neostriatum radiomic features as imaging markers for PD progression and response to treatment remains to be further investigated.

In conclusion, neostriatum radiomics signatures based on T2W images in this study achieved good diagnostic performance for PD and potentially could serve as a basis for the clinical diagnosis of PD. Moreover, our preliminary results showed the potential of neostriatum radiomic features as imaging markers for PD.

## Data Availability Statement

The datasets generated for this study are available on request to the corresponding author.

## Ethics Statement

The studies involving human participants were reviewed and approved by the Ethics Committee at the First Affiliated Hospital of Jinzhou Medical University. The ethics committee waived the requirement of written informed consent for participation.

## Author Contributions

PL and XZ designed the study. PL, SZ, and FZ collected the raw data. SZ and XZ segmented the ROIs. FZ provided clinical expertise. HW and PL performed the data analysis. PL and SZ wrote the manuscript draft. XZ edited the manuscript and supervised the whole study process. All authors had reviewed this manuscript critically and approved its final submission.

### Conflict of Interest

The authors declare that the research was conducted in the absence of any commercial or financial relationships that could be construed as a potential conflict of interest.
