# Supplementary material for: Parkinson's Disease Diagnosis Using Neostriatum Radiomic Features Based on T2-Weighted Magnetic Resonance Imaging
Source: Front Neurol. 2020 Apr 8;11:248. doi: 10.3389/fneur.2020.00248 (PMC7156586; doi:10.3389/fneur.2020.00248)
Supplement: Supplementary file 1 [file Table_1.DOCX]

**Table 1: Outcome of the consensus between the two neuroradiologists (SZ and XZ) wherever there was a disagreement in the image slice selection**

| **Index** | **Participant No.^a^** | **CN/PU** | **Slice No. selected by SZ** | **Slice No. selected by XZ** | **Outcome of the consensus^b^** |
| --- | --- | --- | --- | --- | --- |
| 1 | HC_#28 | PU | #11 | #12 | #11, SZ |
| 2 | HC_#41 | CN | #11 | #10 | #10, XZ |
| 3 | HC_#46 | CN | #11 | #10 | #10, XZ |
| 4 | HC_#60 | CN | #10 | #11 | #11, XZ |
| 5 | PD_#3 | PU | #11 | #10 | #11, SZ |
| 6 | PD_#7 | PU | #11 | #12 | #12, XZ |
| 7 | PD_#28 | CN | #10 | #11 | #11, XZ |
| 8 | PD_#29 | CN | #12 | #11 | #11, SZ |
| 9 | PD_#48 | CN | #11 | #10 | #11, SZ |
| 10 | PD_#48 | PU | #12 | #13 | #13, XZ |
| 11 | PD_#55 | CN | #11 | #12 | #12, XZ |

^a^There were 69 PD patients and 69 healthy controls, thus they were respectively numbered from 1# to 69#.

^b^the final selected slice No. and whose original choices was finally selected.
